# An Investigation of the Antioxidant Capacity in Extracts from *Moringa oleifera* Plants Grown in Jamaica

**DOI:** 10.3390/plants6040048

**Published:** 2017-10-23

**Authors:** Racquel J. Wright, Ken S. Lee, Hyacinth I. Hyacinth, Jacqueline M. Hibbert, Marvin E. Reid, Andrew O. Wheatley, Helen N. Asemota

**Affiliations:** 1 Biotechnology Centre, University of the West Indies, Mona Kingston 8, Jamaica; racq.wright@gmail.com (R.J.W.); awheatley@mset.gov.jm (A.O.W.); 2 Caribbean Institute for Health Research, University of the West Indies, Mona Kingston 8, Jamaica; marvin.reid@uwimona.edu.jm; 3 Department of Chemistry and Biochemistry, Jackson State University, Jackson, MS 39217, USA; ken.s.lee@jsums.edu; 4 Department of Pediatrics, Aflac Cancer and Blood Disorder Center, Children’s Healthcare of Atlanta and Emory University, Atlanta, GA 30322, USA; hhyacinth@emory.edu; 5 Department of Microbiology, Biochemistry and Immunology, Morehouse School of Medicine, 720 Westview Drive SW, Atlanta, GA 30310, USA; jhibbert@msm.edu; 6 Biochemistry Section, Department of Basic Medical Sciences, University of the West Indies, Mona Kingston 8, Jamaica

**Keywords:** *Moringa oleifera*, DPPH, antioxidant activity, oxidative stress, sickle cell anemia

## Abstract

*Moringa oleifera* trees grow well in Jamaica and their parts are popularly used locally for various purposes and ailments. Antioxidant activities in *Moringa oleifera* samples from different parts of the world have different ranges. This study was initiated to determine the antioxidant activity of Moringa oleifera grown in Jamaica. Dried and milled Moringa oleifera leaves were extracted with ethanol/water (4:1) followed by a series of liquid–liquid extractions. The antioxidant capacities of all fractions were tested using a 2,2-diphenyl-1-picrylhydrazyl (DPPH) assay. IC_50_ values (the amount of antioxidant needed to reduce 50% of DPPH) were then determined and values for the extracts ranged from 177 to 4458 μg/mL. Extracts prepared using polar solvents had significantly higher antioxidant capacities than others and may have clinical applications in any disease characterized by a chronic state of oxidative stress, such as sickle cell anemia. Further work will involve the assessment of these extracts in a sickle cell model of oxidative stress.

## 1. Introduction

The *Moringa oleifera* plant, which is also known as “Marengue” in Jamaica, is one of the 13 species in the *Moringa* genus. Other species include *Moringa stenopetala* and *Moringa ovalifolia*. *Moringa oleifera* is also called the Horseradish tree, and like horseradish it possesses a taproot system that supports the umbrella-like canopy of the trunk, leaves, and branches [1,2]. The leaves are tripinnate and grow with a fragile feather-like drooping crown. Fragrant flowers grow as spreading auxillary panicles and are yellowish white, and when fertilized produce pods resembling that of the common bean (*Phaseolus vulgaris*), commonly referred to as string beans or snap beans. Similar to stringbeans, the pods are initially green; however, as *Moringa* pods mature, they become brown and thicker [2]. In Jamaica, flowers and consequently pods are produced at an increased rate during the rainy season, although flowering and fruiting occurs throughout the year. The plant seems to prefer the drier climate of the southern areas in Jamaica but also grows in the north [3]. Several non-governmental organizations including the Food and Agricultural Organization (FAO), Educational Concerns for Hunger Organization (ECHO), Church World Service, and Trees for Life have endorsed *Moringa* as a nutritional gold mine for tropical areas due to its nutritional content and its ability to grow in tropical and drought affected areas [4,5].

*Moringa oleifera* is native to the Himalayas and grows well in sub-tropical and tropical regions of the world. It is widely used in ethnobotany and is thought to cure a variety of diseases [4]. *Moringa* has long been a part of Ayurvedic medicine in India and is understandably referred to as the “Miracle Tree”. All parts of the *Moringa* plant are edible, with leaves and pods used most frequently. The leaves of the plant are utilized as a nutritional supplement, are thought to boost the immune system as well as energy levels, and are known to have anti-inflammatory as well as antioxidant properties. In Jamaica, *Moringa* is used in the preparation of hot and cold beverages (teas and juices) and as meals in many households. The interest in *Moringa* started in the 2000s, and this interest continues today. Various investigations have shown that *Moringa* contains antioxidants [4,6,7,8]. Antioxidants are useful in the management of oxidative stress in the body [9]. *Moringa* could potentially be used to improve the clinical condition of persons with oxidative stress conditions such as sickle cell anemia (SCA). Oxidative stress is the result of an imbalance between reactive oxidative species (ROS) and antioxidant components in the body. ROS can potentially damage cells in the body, destabilizing the cell integrity by reacting with cellular components [9]. Antioxidant components are designed to reduce ROS [9]. The body produces ROS as a part of its normal metabolic processes and consequently has biological mechanisms to counteract oxidation [10].

In healthy people, the antioxidant system usually restores balance easily by reducing ROS when formed [9]. People with SCA experience a relatively higher oxidant load due to factors including the increased frequency of erythrocyte destruction (which results in excessive free heme, a potential oxidant). People with SCA also seem to have an increased metabolic rate, which in turn increases the production of oxidants. This increased oxidant production results in an imbalance of oxidants compared to antioxidants causing oxidative stress [10,11]. Sickle cell anemics therefore have higher levels of ROS due to the inability of their antioxidant system to compensate for the abnormal free heme plasma levels. This results in inflammation and chronic organ damage [9,10].

In Jamaica, 1 in 300 persons are estimated to have SCA in Jamaica. People with SCA experience a variety of symptoms, the consequences of which could be serious and expensive for Jamaicans. Sickle cell anemics often experience sickling crises, for which the disease is named. Leg ulcers, splenomegaly, stroke, pulmonary hypertension, and other conditions may also occur. Inflammation is an underlying symptom associated with many conditions.

People with SCA are treated symptomatically. Symptoms include pain and infections, which are treated with analgesics and antibiotics, respectively. SCA is prevalent in African and Caribbean nations, developing countries with limited resources. *Moringa* is a tropical plant that grows easily in these countries and is therefore readily accessible [4]. The use of the plant would be a cost-effective way of combating SCA by reducing oxidative stress. There appears to be no evidence in the literature of antioxidant activity testing of the Jamaican grown *Moringa* plant. This study was initiated to determine the antioxidant activity of *Moringa* plants grown in Jamaica.

## 2. Results

**Figure 1:**
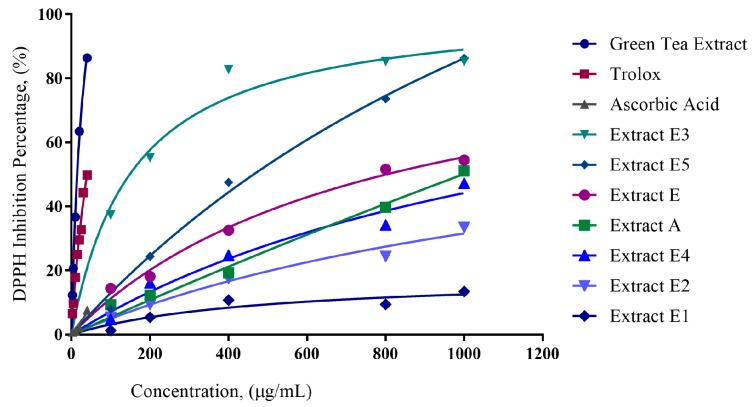
DPPH reduction percentage of Trolox, ascorbic acid, green tea, and the *Moringa oleifera* leaf extracts A, E, E1–5 after 30 minutes. DPPH Reduction Percentage gradients for extracts E3 and E5 (0.147; 0.082) were higher compared with the other extracts, particularly E1 and E2 (0.011; 0.029). E5 and E3 had gradients over 3–7 times greater than E2 and E1, respectively.

Initial extraction with ethanol (E) resulted in 37% recovery. Further fractionation using various solvents, namely hexane, chloroform, butanol, and water was performed. In relation to the ethanol extract, the percentage recovery for the solvent extracts was as follows: hexane-E1 (48%), chloroform-E2 (1.75%), butanol-E3 (9.46%), and water-E5 (18.26%). DPPH reduction percentages are shown in Figure 1. The slope of the DPPH reduction percentage plot was used as an indicator of antioxidant capacity. *Moringa* extracts E3 and E5 have higher slopes compared to E1 and E2 (Figure 1); therefore, E3 and E5 have higher antioxidant capacities compared to E1 and E2.

**Figure 2:**
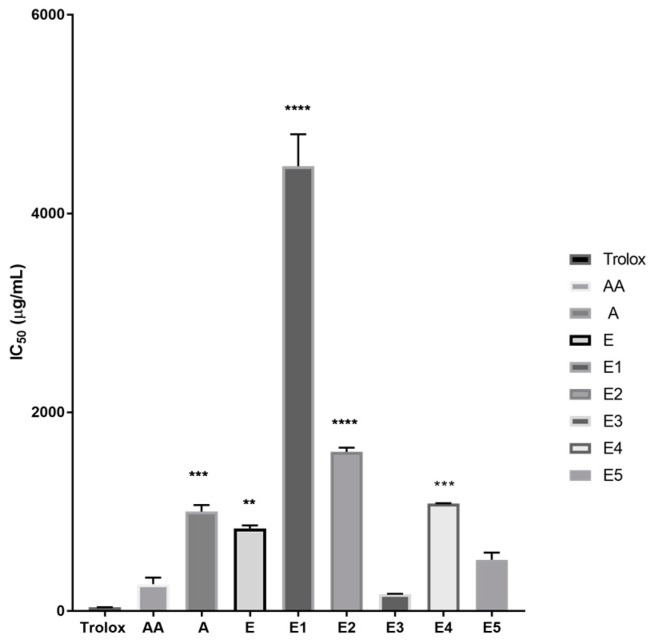
IC_50_ values of ascorbic acid (AA) compared with Trolox and *Moringa* leaf extracts A, E, and E1–E5. Statistical significance was calculated using one-way analysis of variance (ANOVA) followed by Dunnett’s post-hoc test using GraphPad Prism statistical software; values of *p* < 0.05 were considered statistically significant. Significant values were denoted as follows: ** *p* ≤ 0.01; *** *p* ≤ 0.001; **** *p* ≤ 0.0001. Ascorbic acid had an IC_50_ value of 272 µg/mL. Extracts E2 and E1 had IC_50_ values of 1604 µg/mL and 4477 µg/mL respectively, while extracts E, E3, E4, E5, and A had approximately 2–6 times lower IC_50_ values of 833 µg/mL, 173 µg/mL, 1085 µg/mL, 517 µg/mL, and 1003 µg/mL, respectively.

IC_50_ values, representing the sample concentration at which 50% of the DPPH radical has been reduced, were calculated from the DPPH reduction percentages. There is an inverse relation between IC_50_ values and antioxidant activities; this means that lower IC_50_ values indicate a higher antioxidant capacity. Extracts E2 and E1 had IC_50_ values of 1604 µg/mL and 4477 µg/mL, respectively, while extracts E, E3, E4, E5, and A had IC_50_ values of 832.8 µg/mL, 172.6 µg/mL, 1085 µg/mL, 516.9 µg/mL, and 1003 µg/mL, respectively. Based on the graph (Figure 1) showing the percentages of DPPH reduction, extracts E3 and E5 possessed more effective antioxidative capacity compared to the other extracts. Figure 2 also shows that the IC_50_ values of extracts E3 and E5 were lower compared to the other extracts.

## 3. Discussion

Antioxidant activity of *Moringa oleifera* leaf extracts from Jamaica was assessed using the 2,2-diphenyl-1-picrylhydrazyl (DPPH) assay [6,12]. This seems to be the first study of antioxidant activity using *Moringa* sourced from Jamaica, as the literature tends to lack any such information. It should be noted that these extracts were produced from a direct ethanolic extraction of the *Moringa* leaves. Typically, when antioxidant principles are being extracted from samples or when antioxidant tests are being conducted, an alcoholic solvent, aqueous solvent, or a combination of both is used as the extracting agent [6,13,14,15].

The crude extract E was sequentially extracted using solvents of different polarities. This allowed for the sequential separation of compounds based on the affinity to the solvent. This further subdivision of extracts provided a clearer picture as to which solvents have the greater capacity to accumulate antioxidants as well as concentrating the antioxidant extracts to smaller fractions. Extracts E1 and E2 were produced using hexane and chloroform, respectively. Extracts E3 and E5 were produced using polar solvents, namely butanol and water, respectively. In Figure 1, the DPPH reduction percentage was calculated for samples and controls.

The IC_50_ values for each sample was calculated to show the concentration of sample needed to reduce 50% of the DPPH in the assay. Low IC_50_ values correspond to high antioxidant activity. The IC_50_ values show that E3 < E5 < E < A < E4 < E2 < E1 (Figure 2). Both E1 and E2 showed lower antioxidant activities than other extracts, with E1 being the lowest. This could be explained by the fact that hexane was the most non-polar solvent used in the experiment. Antioxidant compounds are known to accumulate in polar solvents and it is understandable that they would not be present in substantial amounts in an extract produced from hexane. Chloroform is more polar than hexane, so the increased antioxidant activity seen in extract E2, compared with extract E1 (hexane), is expected [16,17]. This data also suggests that liquid–liquid extraction of crude extract E with low polarity solvents, hexane and chloroform, was effective in separating fractions with high antioxidant potential, thus producing a more concentrated antioxidant fraction (the residual fraction after extraction with chloroform). This fraction when extracted further produced extracts E3 and E5, which had higher antioxidant capacity compared to the extracts.

Extract E3 had the highest antioxidant activity since it had the lowest IC_50_ value. It is notable that extract E3 possessed a similar IC_50_ value to ascorbic acid (vitamin C), a known antioxidant, which suggests that extract E3 could be a valuable antioxidant. Extract E had a lower antioxidant activity than extracts E3 and E5 based on the IC_50_ value; however, it still had a good antioxidant activity compared with ascorbic acid. These results suggest that the components responsible for antioxidant activity in *Moringa* leaves are polar compounds. The values for *Moringa* compared with the standard Trolox were not an exact match compared with values found in the literature; however, when the values were normalized as a ratio, the values correlated with results obtained by Chumark et al. (2008) [18]. Chumark et al. also showed that Trolox has a lower IC_50_ value compared to the *Moringa* extract, with *Moringa* being 35 times less potent than Trolox. In fact, it seems that the Jamaican *Moringa* had comparatively better antioxidant activity, as this study showed *Moringa* was only four times less potent than Trolox. At the time of this study, *Moringa* could be found in only a few regions of the island, and the material analyzed was from a single region [3]. Further studies are needed to test the *Moringa* leaves from other regions in Jamaica extracted under the same conditions to determine if the antioxidant activity differs from parish to parish, possibly depending on the nature of the soil. All leaves used in this assay were harvested at the same time. Researchers in Pakistan, Iqbal and Bhanger (2006), assessed the variations in the antioxidant activity of *Moringa* leaves in different seasons and sample locations and found that the antioxidant activity varied based on season and locations of the plants from which the samples were obtained [19]. However, it is important to note that Pakistan has a larger variation of climatic conditions compared to Jamaica. It is possible that the plants were affected by the cooler and/or drier conditions experienced there. In Jamaica, given the low variability in climatic conditions, it is possible that the Jamaican plants would not have significant differences in antioxidant activities based on the location and time of harvest. Additionally, it is also possible that Jamaican *Moringa* has adapted over the years to the moderate climate experienced in Jamaica compared with Pakistan’s climate. Although the antioxidant activities of Jamaican *Moringa* leaves should be independent of the harvesting locations, this information is not known. This data would be significant if *Moringa* were to be exploited commercially. It may also be useful to investigate antioxidant activities of *Moringa* plants throughout the Caribbean, where the climatic conditions are generally similar but the nature of the soil may vary.

Siddharaj and Becker (2003) have shown that different agroclimatic conditions can result in different antioxidant capacities [6]. In his results, it was seen that India had the best antioxidant capacity compared to samples taken in Central America (Nicaragua) and Africa. This study, however, did not focus on the soil quality, which is another factor that could affect the quality of bioactive principles in plants. Forster and colleagues (2015) assessed the impact of sulfur (a soil factor) compared to water availability. In the field, water availability would typically be determined by rainfall, which is a climatic factor [20]. This study showed that the availability of sulfur had a positive impact on the presence of the beneficial bioactive principles in *Moringa*. There seems to be no direct study yet done on the effect of other soil factors such as pH, soil nutrient levels, and soil type on the antioxidant capacity in *Moringa* leaves. The studies mentioned above however, seem to allude to the premise that these factors could be responsible for differences between the antioxidant activity seen in different countries.

It should also be noted that the genotype of *Moringa* in Jamaica has not been elucidated. There is therefore no concrete evidence to indicate whether Jamaican *Moringa* is a different cultivar compared to those in Asia or Africa. *Moringa* was initially introduced to Jamaica in 1784; however, Asian and African migrants have entered Jamaica over the years, and it is quite possible that these immigrants may have brought different varieties or cultivars of *Moringa oleifera*. This means that there could be several cultivars of *Moringa oleifera* present in Jamaica as well. For example, a variety with completely white flowers and a different flavor profile has been noted in Jamaica.

Ndhlala and colleagues (2014) analyzed 13 *Moringa* cultivars from four regions (Thailand, Taiwan, United States of America, and South Africa) for antioxidant capacity. The data showed that the samples from Thailand had the best antioxidant capacity, whereas samples from South Africa (Silver Hill) had the lowest activity [21]. Ndhlala and others (2014) postulated among other things that climatic/environmental differences between the regions could be responsible for the variations seen [21]. *Moringa* has been present in Thailand for several decades, and it is possible that the antioxidant profile is due to the plant's adaptation to its environs [21]. Both Jamaica and Thailand have tropical climates; similarly, the environmental conditions under which the Jamaican *Moringa* plant is grown could have a positive effect on its antioxidant capacity. A comparison between samples from different agroclimatic origins would be beneficial to determine if this postulation is a valid claim. Analyses of soil factors would also be beneficial in providing a broader picture of the impact of environmental conditions on antioxidant capacity in *Moringa oleifera*.

## 4. Methods and Materials

### 4.1. Reagents

Methanol (HPLC grade), ethanol, butanol, hexane, and chloroform (ACS grade), ascorbic acid, and 2,2-diphenyl-1-picrylhydrazyl (DPPH) free radical were obtained from Sigma-Aldrich (Saint Louis, Missouri, USA). Green tea extract (pre-standardized to 50% epigallocatechin gallate) was obtained from HerbStoreUS (Walnut, CA, USA).

### 4.2. Extraction of Moringa oleifera Leaves

**Figure 3:**
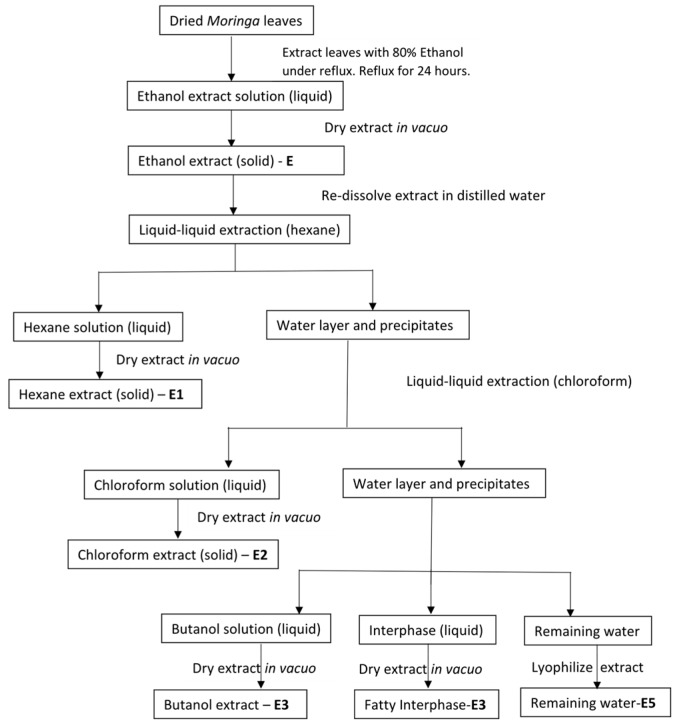
Schematic diagram of the ethanolic extraction process of *Moringa oleifera* leaves.

*Moringa oleifera* leaves were prepared according to the method in Luo et al. (2011) and Oyugi et al. (2009) with modifications [14,22]. The leaves were harvested, rinsed with clean water, and air-dried at room temperature overnight. The leaves were then placed in an oven at 40 °C and left until completely dry. The leaves were milled into a fine powder. The milled leaf samples (20 g) were subjected to soxhlet extraction using 80% ethanol: water mixture (250 mL). The resulting solution was dried in vacuo and stored at 4 °C until further analysis. This extract was called E. Different fractions were obtained by sequential washing with different solvents (Figure 3). Extract E was further purified by washing with non-polar and polar solvents. The dried extract E was re-suspended in 250 mL of water. This aqueous extract was subsequently extracted with 250 mL of hexane, chloroform, and butanol, respectively. The respective solvent was decanted after each extraction and dried in vacuo. Extracts were labeled as follows: E1: hexane; E2: chloroform; E3: butanol; E4: interphase between E5 and E3; E5: remaining water fraction. For the production of Sample A, double distilled water was added to the milled leaves, which were subsequently heated for 24 h with constant stirring. The decoction was filtered and the filtrate lyophilized. All samples were stored at 4 °C until further analysis.

### 4.3. DPPH Radical Scavenging Assay

The antioxidant capacity of the *Moringa oleifera* leaf extracts were analyzed for reductive capacity using the 2,2-diphenyl-1-picrylhydrazyl (DPPH) assay [6,12]. DPPH is a stable free radical that absorbs strongly at 517 nm. When exposed to an antioxidant, it degrades to a light-yellow color that does not absorb at 517 nm. Therefore, the antioxidant activity of a substance can be analyzed based on the how strongly DPPH is absorbed at 517 nm. This assay was done using Trolox, a vitamin E analogue with well-documented antioxidant activity, as the main control for comparison with the extracts. Two additional controls, ascorbic acid and green tea extract, known chemical and herbal antioxidants, respectively, were used to compare the extracts’ antioxidant capacities as they are typical antioxidants found in nature and are weaker than Trolox [15]. The green tea extract was pre-standardized to contain 50% epigallocatechin gallate, the main antioxidant in green tea.

#### 4.3.1. Experimental Procedure

Controls and extracts E and E1–E5 were diluted to appropriate concentrations in methanol to ascertain the antioxidant capacity of all compounds in a linear range. The extracts were diluted to concentrations of 100, 200, 400, 800, and 1000 µg/mL. The standard (Trolox) was diluted to concentrations of 2.5, 5, 10, 15, 20, 25, 30, and 40 µg/mL. The controls (ascorbic acid and green tea extract) were diluted to concentrations of 2.5, 5, 10, 20, and 40 µg/mL; 0.1 mM DPPH in methanol was used and prepared fresh daily.

Controls and extracts were analyzed according to the method of Williams et al. (2006) with some modifications. The controls and extracts (40 µL each) were reacted with DPPH (200 µL each) in 96-well microplates for 30 min in the dark. A blank using methanol, in place of the sample, was also prepared and incubated with the samples. After incubation, the absorbance of samples was read using a Spectromax Pro 250 (Molecular Devices, Sunnnydale Ca.) microplate reader at 517 nm.

The mean optical density of the sample was used to calculate the DPPH reduction (inhibition), which is the percentage of DPPH that was neutralized by the antioxidants present in the added samples.

The equation below was used to calculate this percentage:

The DPPH reduction percentage is calculated as follows:

[(A_O_ − A_S_)/Ao] × 100 (1)

where A_O_ is the blank absorbance, and A_S_ is the sample

The results were tabulated and presented in graphical format.

The IC_50_ values were calculated from the DPPH reduction percentages and referred to the concentration of sample (in µg/mL) required to reduce 50% of the DPPH present in the assay.

#### 4.3.2. Statistical Analysis

Mean ± SEM was calculated from replicates of three or more for each sample. Significant differences between the values were calculated using Dunnett’s post-hoc test with GraphPad Prism 5.0. Values of *p* < 0.05 was considered statistically significant.
